# Erythrocytapheresis in Children and Young Adults with Hemoglobinopathies and Iron Overload in Need of Iron Chelation Therapy

**DOI:** 10.3390/jcm12196287

**Published:** 2023-09-29

**Authors:** Jessica van Hattem, Philip Maes, Tonya Marianne Esterhuizen, Ann Devos, Martin Ruppert, Jaques van Heerden

**Affiliations:** 1 Department of Paediatrics and Child Health, Antwerp University Hospital, 2650 Antwerp, Belgium; 2 Department of Paediatric Haematology and Oncology, Antwerp University Hospital, 2650 Antwerp, Belgium; philip.maes@uza.be (P.M.); ann.devos@uza.be (A.D.); jaques.vanheerden@uza.be (J.v.H.); 3 Division of Epidemiology and Biostatistics, Faculty of Medicine and Health Sciences, Stellenbosch University, Cape Town 7505, South Africa; tonyae@sun.ac.za; 4 Department of Abdominal, Paediatric and Reconstructive Surgery, Antwerp University Hospital, 2650 Antwerp, Belgium; martin.ruppert@uza.be

**Keywords:** erythrocytapheresis, hemoglobinopathies, β-thalassemia major, sickle cell disease, children, transfusion iron overload, chelation therapy

## Abstract

Limited data regarding erythrocytapheresis in children, adolescents, and young adults have been published. The aim of this study was to evaluate erythrocytapheresis, either as a standalone therapy or in combination with iron chelation therapy, in children and young adults with hemoglobinopathies in whom current iron chelation therapy is not sufficient in decreasing the iron overload during management. We retrospectively analysed erythrocytapheresis in 19 patients with hemoglobinopathies in need of iron chelation therapy diagnosed with sickle cell disease (SCD) or β-thalassemia major. Patients were divided into (1) a case cohort who received erythrocytapheresis alone or in combination with iron chelation therapy and (2) a control cohort who received oral iron chelation therapy alone. Serum ferritin and haemoglobin levels were compared at five different time points over a one-year period. In the erythrocytapheresis cohort, there was a significant decrease in serum ferritin (*p* < 0.001). In the iron chelation therapy alone cohort, there was no significant decrease in serum ferritin over time (*p* = 0.156). Comparing the evolution of median serum ferritin between therapy with erythrocytapheresis and iron chelation therapy showed a statistically significant difference (*p* = 0.008). Patients with β-thalassemia major receiving erythrocytapheresis showed a greater reduction in serum ferritin compared to patients without (*p* = 0.036). A difference could not be shown between the erythrocytapheresis and iron chelation single therapies (*p* = 0.100). This study showed an overall significant reduction in serum ferritin in patients with hemoglobinopathies treated with erythrocytapheresis in addition to iron chelation. A clinical, although not statistical, trend of higher haemoglobin levels was maintained. Erythrocytapheresis in paediatric patients with β-thalassemia major was as effective in decreasing ferritin levels as in previously reported studies with SCD. Erythrocytapheresis is a promising therapy for treating and preventing transfusion-related iron overload.

## 1. Introduction

In the treatment of hemoglobinopathies, such as sickle cell disease (SCD) and thalassemia, red blood cell (RBC) transfusions are essential for the management of symptomatic anaemia [1,2]. Repeated transfusions lead to accumulation of iron in soft tissues, resulting in inflammation, susceptibility to infections, end-organ damage and death. These include cardiomyopathy, liver cirrhosis, multiple endocrine abnormalities such as hypothyroidism, growth retardation, hypogonadism, delayed puberty, and diabetes, with an increased risk of developing hepatic malignancies [3]. Although iron chelation therapy is available, an optimal and durable response is not guaranteed [4,5,6]. This may be due to several factors that include non-compliance to therapy, inadequate dosage, drug resistance due to changes in the iron chelator’s target binding sites, variable tissue distribution, or genetic variations that may affect the metabolism, absorption, or excretion of iron and chelating agents [4]. Erythrocytapheresis, an automated method of RBC exchange, has been reported to reduce iron overload in chronically transfused patients. Although a significant number of publications on the use of erythrocytapheresis in disease control in both children and adults diagnosed with SCD have been published, only a limited number of reports utilising erythrocytapheresis in patients with SCD and co-commutant iron overload are available [7,8,9,10]. In patients with SCD, erythrocytapheresis has been applied not only as a chronic transfusion modality in treating SCD complications such as stroke, acute chest syndrome, priapism, dactylitis, and splenic sequestration but to optimise the clinical condition in patients towards haemopoietic stem cell transplantation and the acute indications where an end organ is threatened [11]. These reports, however, focus on the disease pathophysiology rather than regulating iron overload.

Patients with thalassemia are in need of chronic RBC transition therapy and are also at risk of developing iron overload. Erythrocytapheresis has no role in disease regulation in patients with thalassemia, but as in patients with SCD, even fewer reports of erythrocytapheresis for the treatment of iron overload in patients with thalassemia have been published [12]. These reports are limited to adults [12].

The gold standard for evaluating tissue iron overload is a biopsy, but because of its invasive nature, magnetic resonance imaging (MRI) has become the primary outcome measure for clinical trials of iron chelation therapy [4,13]. In children, both methods are logistically problematic and resource-intensive. Therefore, the most efficient and cost-effective method for quantifying iron overload is to determine serum ferritin levels, especially before clinical signs of iron overload appear. It is also an effective method for monitoring iron chelation therapy, even in the presence of factors that affect serum ferritin levels [4].

At the Antwerp University Hospital, a tertiary healthcare facility, erythrocytapheresis has been implemented as a standard of care during the management of iron overload in patients with SCD, who received chronic blood transfusions, and with β-thalassemia major, who are transfusion dependent, to decrease ferritin levels as well as prevent permanent complications of iron overload in these patients. The aim of this study was to evaluate erythrocytapheresis, either as a standalone therapy or in combination with iron chelation therapy, in children and young adults with hemoglobinopathies in whom current iron chelation therapy is not sufficient in decreasing the iron overload during management. Two secondary objectives were (1) to evaluate if erythrocytapheresis has similar benefits in regulating iron overload in both patients with SCD and β-thalassemia major and (2) to evaluate the cost-benefit ratio involved when implementing erythrocytapheresis during management compared to iron chelation therapy alone.

## 2. Materials and Methods

This retrospective study included patients diagnosed with SCD or thalassemia, in need of chronic blood transfusions between 2015 and 2022, who developed iron overload based on serum ferritin levels where iron chelation therapy did not provide effective treatment in decreasing serum ferritin levels. Patients in the study population were of Central African, South East Asian, and Middle Eastern origin. Antwerp serves a large immigrant community, who are in need of significant social support. Newly immigrated patients also present with untreated iron overload from their native countries.

Poor adherence to oral iron-chelation therapy was defined as the patient or caregiver reporting poor adherence, irrespective of the reason, or documented unclaimed medication from pharmacy records.

Patients were divided into two groups: (1) a case cohort who received erythrocytapheresis (irrespective of the duration) alone or in combination with iron chelation therapy and (2) a control cohort who received oral iron chelation therapy alone. Where a sub-analysis was possible, the case cohort was divided into two subgroups: (1) erythrocytapheresis alone and (2) iron chelation therapy in combination with erythrocytapheresis.

All patients commenced iron chelation therapy when ferritin levels reached 1000 μg/L or higher and continued as ferritin levels did not decline sufficiently. In our study, all patients received Deferasirox (DFX), which is an oral iron chelator. Institutional indications for starting erythrocytapheresis are (1) rising ferritin levels despite adequate iron chelation therapy, (2) medical reasons for low compliance to iron chelation, and (3) social factors limiting compliance to iron chelation therapy.

The collected epidemiological data included sex/gender, age, and diagnosis. Laboratory test results were also collected from the patient’s file. These blood examinations were performed during the standard follow-up consultation for patients treated with iron chelation therapy every 1 to 3 months for patients treated with erythrocytapheresis, biweekly to monthly, depending on the treatment frequency. Blood examinations were performed using the following devices: for biochemical evaluations, the Attelica Solution Immunoassay and Clinical Chemistry Analyzer ^®^ by Siemens^®^ (Munchen, Germany) and for the full blood counts, the XN 9100 by Sysmex^®^ (Kobe, Hyogo, Japan). 

To evaluate the status of iron overload and the maintenance of stable RBC parameters, the following laboratory results were analysed: haemoglobin (Hb), haematocrit (Hct), reticulocyte count (Ret), serum ferritin (Fe), and lactate dehydrogenase (LDH) as potential indicators. To evaluate end-organ damage, aspartate aminotransferase (AST), alanine aminotransferase (ALT), gamma-glutamyl transferase (GGT), alkaline phosphatase (ALP), total serum bilirubin (TBil), serum conjugated bilirubin (cBil), and serum unconjugated bilirubin (uBil) were selected as indicators.

Data to evaluate erythrocytapheresis therapy long-term outcomes were collected at five different time points (at the start of either erythrocytapheresis or DFX alone therapy, after 1 month, after 3 months, 6 months, 9 months, and 1 year) from the start of erythrocytapheresis therapy until one year post commencing erythrocytapheresis therapy. Ferritin and Hb were collected at five standardised time points to prevent confounding by short-term outcomes of acute events that could affect acute phase reactants. Similarly, Hb values were evaluated over the long-term trend, thereby preventing confounding by fluctuations due to intermittent red blood cell transfusions.

In the absence of clinical symptoms or indications, no echocardiograms and advanced imaging, using MRI or Ferriscan to detect cardiac or liver iron overload, were performed during the follow-up period of one year.

### 2.1. The Erythrocytapheresis Protocol

Long-term venous access is established in all patients utilising one of two methods: (1) two high-pressure intravenous 14-gauge catheters (Port-a-Cath^®^, Smiths Medical, Minnesota, USA), one placed in the subclavian vein, and one placed in the inguinal vein or (2) a surgical arteriovenous fistula is created in the cubital fossa.

Erythrocytapheresis was performed with the Spectra Optia^®^ system (Caridian BCT, Inc., Colorado, USA). The erythrocytapheresis volume was calculated using the patient’s height, weight, and sex. Before the start of erythrocytapheresis and at the end, a blood examination is performed.

The total procedure takes approximately three to four hours while the patient is monitored for cardiovascular and respiratory parameters. Patients receive erythrocytapheresis every 2 to 4 weeks based on the ferritin levels. No international guidelines exist to guide this frequency of treatments.

### 2.2. Statistical Analysis

Data were analysed using IBM SPSS version 28 (IBM Corporation, USA). A *p*-value of <0.05 was considered statistically significant.

Data were expressed as median with minimum and maximum and quartiles (25% and 75% percentiles) due to the small study sample and distinctly non-normal distribution of quantitative variables (tested with Kolmogorov–Smirnov tests). Associations between two categorical variables were assessed using Pearson chi-square analysis or Fisher’s exact two-sided tests as appropriate. One-way ANOVA was used to analyse the trends of association between categorical and continuous normally distributed variables. The Mann–Whitney U test was used to compare distributions of abnormally distributed variables between two categories. The Kruskal–Wallis test and Friedman test were used to compare medians of more than two categories between and within subjects, respectively.

Some cohorts were too small (less than five) for meaningful calculations and were reported as trends. The single sickle-thalassemia patient was included in all analyses where inclusion criteria were met but was never analysed as a single patient cohort as it does not constitute a representative cohort.

## 3. Results

Nineteen patients were included in the study. There were 11 (57.9%) males and eight (42.1%) females with an M:F ratio of 1:0.7. The median age at the time of the study was 13 years, ranging from one to 27 years. The cohort consisted of 10 (52.6%) patients diagnosed with SCD, eight (42.1%) with β-thalassemia major, and one (5.3%) with sickle-thalassemia. The study cohort was divided into two cohorts based on their treatment modalities (see Table 1).

### 3.1. Cohorts

#### 3.1.1. Combined Chelation Therapy and Erythrocytapheresis Cohort

This cohort consisted of 10 (52.6%) patients with six (60.0%) males and four (40.0%) females. Seven (70.0%) patients received concomitant iron chelation therapy and erythrocytapheresis. The remaining three (30.0%) patients stopped with oral iron chelation therapy at the start of erythrocytapheresis. The treatment with erythrocytapheresis had to be stopped during the study period for three patients (30.0%)—two due to venous access problems and one after receiving a haematological stem cell transplantation. The data was robust enough for the three patients to be included in the analysis.

#### 3.1.2. Chelation Therapy Alone Cohort

The second cohort consisted of nine (47.4%) patients, five (55.6%) males, and four (44.4%) females who only received Deferasirox iron chelation therapy.

### 3.2. Outcome According to Indicators

Serum ferritin and haemoglobin values were the only two laboratory parameters that showed changes over time. Other haematological parameters and biochemical investigations to monitor end organ damage were not statistically significant.

#### 3.2.1. Ferritin

**Figure 1:**
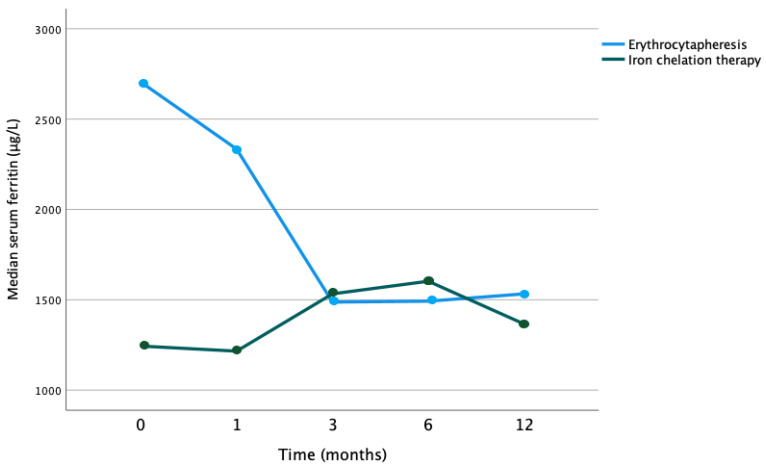
Evolution of median serum ferritin values over time according to therapy with erythrocytapheresis or iron chelation therapy (*p* = 0.008).

In the combined cohort, there was a significant decrease in serum ferritin (*p* < 0.001). In the iron chelation alone cohort, there was no significant decrease in serum ferritin over time (*p* = 0.156). Comparing the evolution of the median serum ferritin values between the cohort receiving erythrocytapheresis and those without showed a statistically significant difference (*p =* 0.008) (see Figure 1).

**Figure 2:**
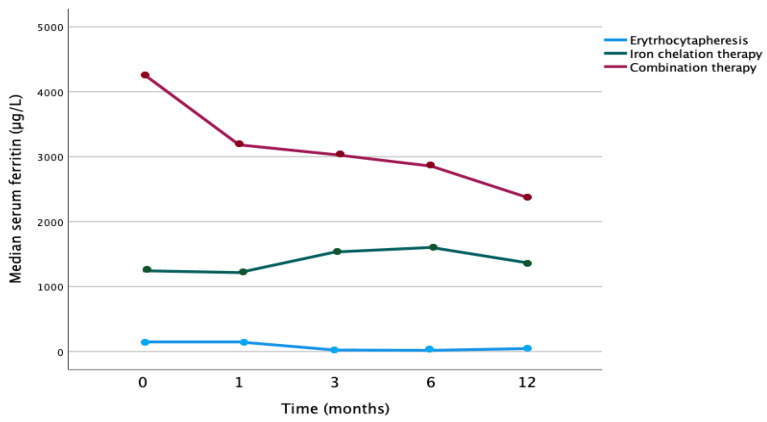
Evolution of median serum ferritin over time by therapy with erythrocytapheresis only, iron chelation only, or combination therapy (*p* = 0.012).

Patients receiving erythrocytapheresis alone showed a decrease in median serum ferritin values of 102 μg/L (IQR: 1046—39 μg/L), and patients receiving erythrocytapheresis in combination with iron chelation showed a greater median decrease of 2195 μg/L (IQR 2746—827 μg/L) (*p* = 0.067). The decrease of serum ferritin values in the combination therapy cohort was greater compared to both the single therapy cohorts with either erythrocytapheresis alone or iron chelation therapy alone (*p* = 0.012) (see Figure 2).

There was no significant change in the reduction in serum ferritin values when comparing the patients in the two single therapy cohorts (*p* = 0.100).

#### 3.2.2. Haemoglobin

**Figure 3:**
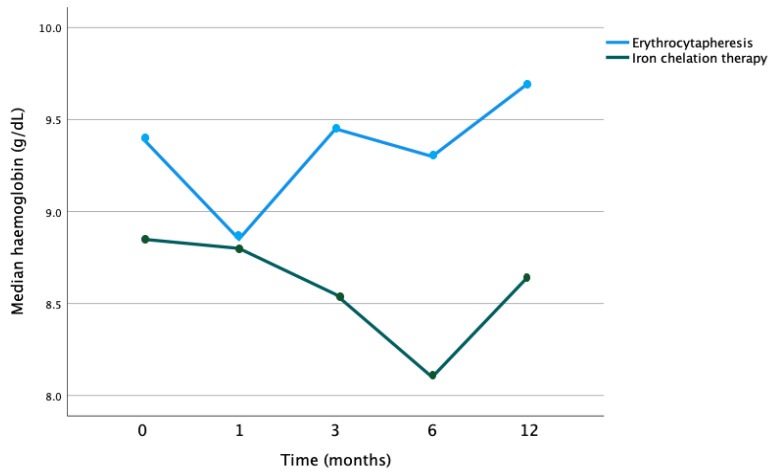
Evolution of median serum ferritin over time by therapy with erythrocytapheresis or iron chelation (*p* = 0.079).

Patients treated with erythrocytapheresis showed higher levels of haemoglobin with less fluctuation over time compared to patients treated with iron chelation (*p* = 0.079). Patients receiving only iron chelation therapy showed unstable haemoglobin levels evolution over time with lower baseline levels (Figure 3).

### 3.3. Outcomes According to Diagnosis

#### 3.3.1. Patients Diagnosed with Sickle-Cell Disease

**Figure 4:**
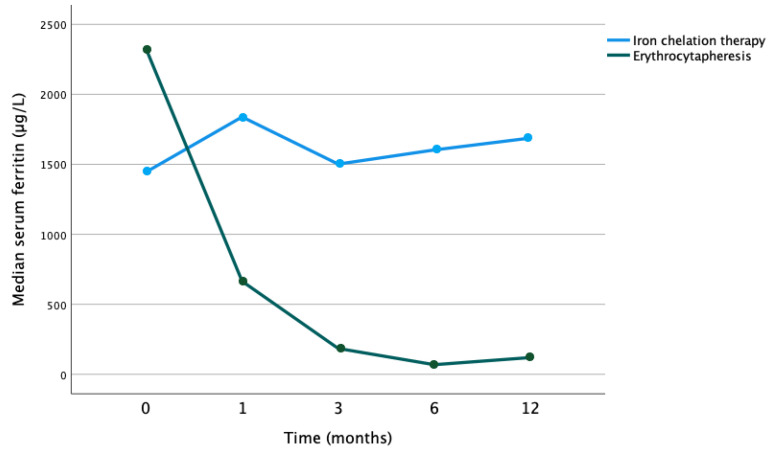
Evolution of median serum ferritin in sickle cell patients by therapy with erythrocytapheresis or iron chelation (*p* = 0.017).

Patients with SCD receiving erythrocytapheresis (combined and single therapy erythrocytapheresis together) showed a greater reduction in serum ferritin (*p* = 0.017) compared to patients with sickle cell disease receiving iron chelation alone (Figure 4). A trend of reduction in serum ferritin, though statistically insignificant, was noted (*p* = 1.000) in patients with SCD only with erythrocytapheresis.

#### 3.3.2. Patients Diagnosed with β-Thalassemia Major

**Figure 5:**
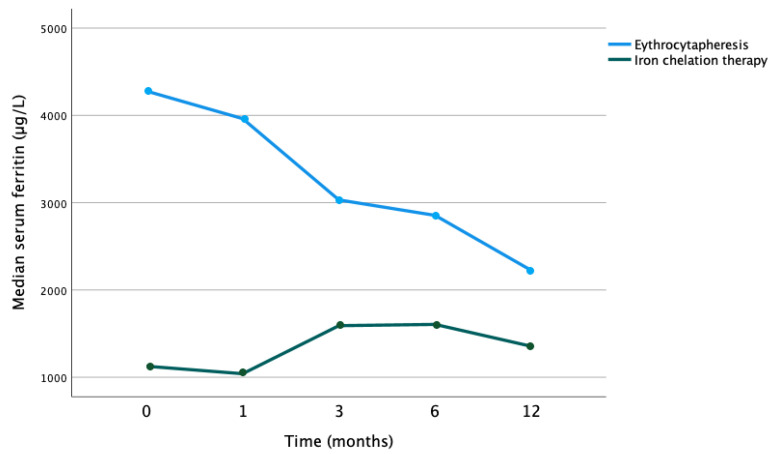
Evolution of median serum ferritin in patients with β-thalassemia major (*p* = 0.036).

Patients with β-thalassemia receiving erythrocytapheresis (combined and single therapy erythrocytapheresis together) showed a greater reduction in serum ferritin compared to patients receiving only iron chelation therapy (*p* = 0.036) (Figure 5).

### 3.4. Outcomes According to Treatment Modality

#### 3.4.1. Erythrocytapheresis Therapy

**Figure 6:**
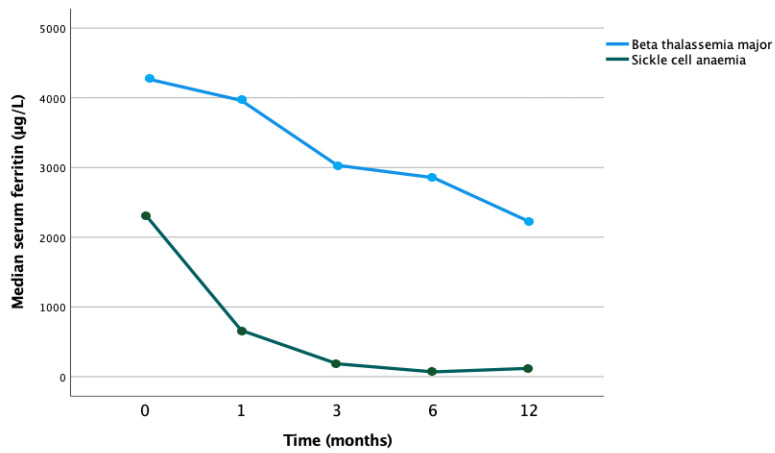
Evolution of median serum ferritin by type of hemoglobinopathy in patients treated with erythrocytapheresis (*p* < 0.001).

In patients with SCD and β-thalassemia major receiving erythrocytapheresis, a significant reduction in serum ferritin in both cohorts was demonstrated (*p* < 0.001) (Figure 6).

#### 3.4.2. Iron Chelation Therapy

When comparing the patients with SCD to patients with β-thalassemia major receiving iron chelation therapy, no significant differences in the reduction in serum ferritin levels were found in both cohorts (*p* = 0.667).

### 3.5. Cost Analysis

A cost analysis was performed comparing the cost of iron chelation therapy with erythrocytapheresis (see Table 2). The total cost for iron chelation therapy ranges between USD 285.40–890.40 depending on the dosage of Deferasirox. The cost of a single erythrocytapheresis is USD 309.20, which is comparable to the lowest dosage of Deferasirox. Prior to initiating erythrocytapheresis therapy, there is an initial one-time cost for the surgical placement of venous access. This is USD 1012.00 for two Port-a-Cath^®^ and USD 801.00 for an arteriovenous fistula.

## 4. Discussion

Erythrocytapheresis, in combination with iron chelating therapy, provides a significant reduction in serum ferritin levels in patients with hemoglobinopathies and maintains the haemoglobin levels at a more stable level. Transfusion-related iron overload is a global challenge in patients with hemoglobinopathies receiving chronic transfusions, and they have potential long-term complications due to iron storage in tissues [14]. Although symptoms or complications of iron overload occur over several years of excessive iron intake or conditions that cause increased iron absorption, timely and efficient intervention remains important in the prevention of long-term effects [15]. Oral iron chelation therapy has shown good results in the treatment of iron accumulation; however, it has not achieved optimal results due to factors such as poor tolerance and treatment compliance [4].

A limited number of studies evaluating erythrocytapheresis for the treatment of iron overload in chronically transfused patients with SCD concluded that erythrocytapheresis is a safe and efficient technique [8,9,10,11]. Therapy with erythrocytapheresis achieved a significant decrease or stabilization of serum ferritin with or without Deferoxamine iron chelation therapy [7,8,9,10]. In our study, we could not prove that erythrocytapheresis alone could significantly reduce serum ferritin levels compared to iron chelation therapy alone. However, combination therapy was better than single-modality therapy. Hilliard et al. and Singer et al. showed stabilization of serum ferritin in three and four non-chelated patients [9,10], while Kim et al. and Adams et al. showed a significant decrease in serum ferritin in two and three non-chelated patients [7,8] (see Table 2). Similarly, we demonstrated that erythrocytapheresis significantly decreased serum ferritin levels in patients who received concomitant Deferasirox. In this study, three patients showed a decrease in serum ferritin levels when receiving erythrocytapheresis alone, although not statistically significant.

Previous studies all performed erythrocytapheresis in patients with SCD. Our study reports novel findings showing a significant reduction in serum ferritin in paediatric patients with β-thalassemia major treated with erythrocytapheresis. A single study from Wall et al. evaluated erythrocytapheresis in adult patients with β-thalassemia major without benefit [12]. In this study, erythrocytapheresis did not have benefits in treating patients with established iron overload [12]. We postulate that our study did reduce serum ferritin levels, indicating the potential benefits for the treatment and prevention of iron overload in children. This study analysed different parameters concerning iron metabolism. Recent studies reported novel markers of iron metabolism, such as soluble hemojuvelin and hepcidin, that are more sensitive and specific than ferritin [16,17]. As this was a retrospective study, these parameters should be implemented in new studies evaluating the efficacy of erythrocytapheresis in the management of iron overload due to chronic transfusions [16,17].

Importantly, previous studies mentioned several complications in vascular access because children have small vessels. Our study showed optimal vascular access using a Port-a-Cath^®^ system, placing one in the shoulder and one in the groin. Moreover, no complications were reported with the prudent use of this system. Therefore, the additional risks associated with the surgical placement of the catheters to perform erythrocytapheresis as standard therapy for transfusion iron overload are less than the complications resulting from the sequelae of iron overload over time. Previous studies have shown that venous access is a barrier to standardization of erythrocytapheresis [7,8,9,10] that may be eliminated with the Port-a-Cath^®^ system.

There is no international consensus for starting erythrocytapheresis, which explains our varied study population. According to the literature, the inclusion criteria for erythrocytapheresis are high iron loads with/or without end-organ damage, poor chelation use, good venous access, and a history of stroke [7,8,9,10]. Standardizing international criteria for the indication to start erythrocytapheresis should, therefore, be prioritised. The data regarding the social impact of both therapies was not robust enough for evaluation in our study. However, reports on long-term iron chelation have shown variable results, especially with regard to compliance. Therefore, more comparative studies evaluating the quality of life during therapy with erythrocytapheresis and iron chelation therapy are needed. As the follow-up period in this study was only one year, the period to analyse long-term complications of iron overload was insufficient as complications developed over years [16].

A more in-depth cost-benefit analysis is indicated, but as this is a new technique, it is important to evaluate the erythrocytapheresis and iron chelation therapy implications of both singular and combined therapies. Excluding the costs for gaining venous access, the monthly costs for the single therapies are comparable, except if erythrocytapheresis is performed more than once a month. This may be necessary in the initial phase of starting erythrocytapheresis therapy but may become less over time. However, iron chelation therapy doses may need to be escalated when the initial response is inadequate or when the age-related doses increase. These costs become wasted if compliance is poor. In this study, only a single Port-a-Cath^®^ was removed due to infections, but it increases the cost during erythrocytapheresis management. When combination therapy is needed, the costs are naturally higher. The question remains whether either therapy can be discontinued when good control has been achieved. In the case of non-compliance, continuation of erythrocytapheresis seems to be more beneficial. If these two therapies are used to achieve stem cell transplantation, the costs are for a limited period of time.

Considering the added benefit of erythrocytapheresis to lower Hb-S levels in patients diagnosed with SCD to achieve disease control increases the cost-effectiveness of this treatment modality. In patients receiving iron chelation therapy alone, hydroxyurea for disease control also needs to be included in the cost analysis. This increases the cost of oral therapies, increases the number of medications to be taken daily, and increases the burden on adherence.

In contrast to the short-term costs, the costs related to end organ damage are potentially much greater than the monthly costs of disease control. This may also include higher morbidity, mortality, and burden of disease [3]. A further consideration is the economic impact of progressive disease and complications related to iron overload [3,14].

The question remains whether either therapy can be discontinued once good control has been achieved. In the case of non-compliance, continuation of erythrocytapheresis would be potentially more beneficial. If the two therapies are used to achieve stem cell transplantation, the cost is for a limited period of time.

Although this study was conducted in a high-income setting, the risk-benefit ratios, particularly in socioeconomic terms, change when the impact is assessed in low-and middle-income countries, such as Africa and South-East Asia, which have the highest burden of SCD and β-thalassemia major [18,19].

This study was limited because of its retrospective nature and incomplete and limited data, as it was conducted in a single institution. The lack of international standards for the initiation of erythrocytapheresis has added to the diversity in the groups. Outcomes and benefits were defined by trends in haemoglobin and ferritin levels during a 1-year follow-up period; no analysis of long-term complications of iron overload, such as cardiac and liver MRI, was performed. In general, an increased risk of morbidity and mortality is associated with ferritin levels above 2500 ng/mL [4], which partly explains the absence of complications in this study, as chelation therapy was started once ferritin levels reached 1000 ng/mL. International recommendations recommend an initial MRI scan after at least 10 transfusions of 15 mL/kg per transfusion or before reaching 10 transfusions if clinical symptoms are present [4].

We acknowledge a possible inherent bias in the study because of no international standards for the indications of erythrocytapheresis. This study used the objective failure to decrease serum ferritin levels to standardise the indication.

## 5. Conclusions

Erythrocytapheresis is a promising, successful therapy for treating and preventing transfusion-related iron overload, as this study showed a significant decrease in serum ferritin levels in patients treated with erythrocytapheresis and a trend of stable haemoglobin levels. Further, the cost-benefit analysis showed that erythrocytapheresis is feasible to be implemented in the management of iron overload due to chronic transfusion. As there is no standardised technique in children, further studies are needed to optimise erythrocytapheresis in children and extrapolate our findings to a larger population independent from the resource setting.

## Figures and Tables

**Table 1:** Clinical, laboratory, and management characteristics.

Pt.	Sex/ Gender	Age in Years	Diagnosis	Red Blood Cell Transfusion Need	Type of Therapy	Frequency of Pheresis	Initial Ferritin (μg/L)	Ferritin at 12 m (μg/L)	Initial Hb (g/dL)	Hb at 12 m (g/dL)
Case group (erythrocytapheresis alone or in combination with oral chelation therapy)										
1	M	17	Beta	Biweekly	Pheresis and DFX	Every 3 weeks	4279	1533	7.7	8.4
2	M	11	Beta	Biweekly	Pheresis and DFX	Biweekly	3051	2224	8.9	7.6
3	M	14	SCD	Monthly	Pheresis and DFX	Monthly	2360	165	10	11.4
4	F	25	SCD	Monthly	Pheresis and DFX	Monthly	148	46	8.1	9.7
5	F	15	SCD	Monthly	Pheresis	Monthly	5921	2522	11	11.4
6	F	24	SCD	Monthly	Pheresis and DFX	Monthly	114	75	11.4	10.6
7	M	19	SCD	Monthly	Pheresis	Monthly	1071	25	11.4	9.7
8	F	30	SCD	Monthly	Pheresis	Monthly	5631	5219	8.2	8.6
9	M	26	SCD	Monthly	Pheresis and DFX	Monthly	2327	-	9.8	-
10	M	6	Beta	Biweekly	Pheresis and DFX	Biweekly	5401	4300	9	7.7
Control group (oral iron chelation therapy alone)										
11	F	14	Beta	Biweekly	DFX	-	1216	1107	9.2	10
12	M	16	Beta	Monthly	DFX	-	1127	1380	10.6	9.6
13	M	6	Beta	Biweekly	DFX	-	1826	1366	10.8	-
14	M	1	Beta	Biweekly	DFX	-	882	1350	-	7.4
15	F	18	SCD-Beta	Monthly	DFX	-	2622	47	8.5	8.6
16	M	10	SCD	Every 3 weeks	DFX	-	1445	1572	7.7	7.7
17	F	28	SCD	Every 1–2 months	DFX	-	1244	1688	6.6	6.8
18	F	33	SCD	Every 2 months	DFX	-	1523	1902	8.4	8.7
19	M	3	Beta	Biweekly	DFX	-	1077	1355	10.3	9.9

Abbreviations: Pt: patient number; Beta: β-thalassemia major; DFX: Deferasirox; Hb: haemoglobin; SCD: sickle cell disease.

**Table 2:** Cost analysis.

Item	Amount	Price US Dollar
**Oral iron chelation therapy**		
Deferasirox	90 × 90 mg	USD 210.00
	90 × 180 mg	USD 410.00
	90 × 360 mg	USD 815.00
Consultation (per 30 min)	1×	USD 75.40
Total		USD 285.40–890.40
**Erythrocytapheresis**		
Erythrocytapheresis	1×	USD 309.20
Surgical placement venous access		
(1) Port-a-Cath^®^	1×	USD 304.00
	2×	USD 608.00
(2) Venous fistula	1×	USD 398.00
(3) Operation time	1×	USD 403.00

## Data Availability

Data created during this study is available on reasonable request within determinations of privacy laws or ethical restrictions.
